# Supplementary material for: Western red cedar (Thuja plicata) beehives have no impact on honey bee (Apis mellifera) overwintering colony survival or detoxification enzyme expression
Source: PLoS One. 2025 Apr 9;20(4):e0318764. doi: 10.1371/journal.pone.0318764 (PMC11981213; doi:10.1371/journal.pone.0318764)
Supplement: Attachment — Submitted filename: point-by-point.docx [file pone.0318764.s004.docx]

**Point-by-point response to reviews**

Editorial requests:

We have addressed all editorial requests as described below.

1. We have formatted the manuscript headings and subheadings as described in the PLoS One formatting template. We have also removed bolded figure references and renamed the supplementary files “S1 File” and “S2 File”.
2. We have checked the title, author, and affiliation information and it appears to already be adhering to PLoS style guidelines.
3. We have updated the title entered in the submission system to match the manuscript file
4. We have updated the financial disclosure statement to indicate that “The funders had no role in study design, data collection and analysis, decision to publish, or preparation of the manuscript.”
5. We have renamed the “Olivarez” honey bee queen source as “California” to adhere to the anonymization request
6. We have checked our reference list and have no changes to make

Reviewer requests:

Reviewer #1: I always appreciate practically oriented articles, and your contribution is both easy to read and highly understandable. The design of your experiment is well-structured. While there are other applicable physiological indicators for measuring colony stress, I believe that evaluating detoxification enzyme expression is sufficient for a pilot study. Congratulations on presenting an inspiring idea worth pursuing (it would be valuable to test other resin-rich woods, including some types of pine) and for delivering a concise and focused analysis without delving unnecessarily into peripheral topics.

**Author response:** We deeply appreciate the positive view of our manuscript and for recognizing the overall value of the research question.

Reviewer #2: This manuscript presents a well-designed and executed study investigating the effects of western red cedar hive boxes on honey bees. The authors find no evidence of adverse effects at both the colony and molecular levels. While further research is a demand, the study provides valuable preliminary data suggesting that western red cedar is likely a safe material for hives especially that most of the researches used extracts from the cedar wood not the wood itself. The manuscript is suitable for publication after addressing the minor revisions that will be suggested.

**Author response:** Thank you for the favourable review and providing us with some points of feedback. We have revised the manuscript in light of these comments and hope that, with these changes, it is now considered suitable for publication.

1- While Authors selected old-growth, air-dried cedar, they didn't quantify the actual concentration of extractives in the wood. This makes it difficult to determine the true level of exposure. Analyzing the chemical composition of the wood used would strengthen the conclusions.

**Author response:** This is a good suggestion and we agree that such an analysis would strengthen our conclusions; however, we unfortunately are not able to generate the suggested data. As the wood has now been weathered for 1.5 years, the extractive content, if we were to measure it now, would likely be lower than it would have been at the time the bees were first hived in the boxes. We do think that this is an important caveat and have now mentioned it in the first paragraph of the discussion:

*“However, since we did not quantify the levels of known insecticidal compounds within the cedar wood, we cannot comment on the specific intensity of exposure that the bees experienced, nor did we measure an exhaustive list of outcomes (impacts of wood type on behaviors and hive matrices, for example, were not recorded).”*

We note that we already do discuss this topic in more depth in the final paragraph of the discussion, shown below:

*“Although we report only null effects of hive type and conclude that western red cedar is likely a suitable construction material for beehives, we acknowledge that there may be yet untested contexts in which this is not the case. For example, extractive content can vary from batch to batch of lumber, and while we utilized hives derived from old growth trees (which have, on average, a higher extractive concentration [16, 20]), we did not actually analyze the concentrations of bioactive compounds in the wood. There is a wide spectrum of potential concentrations even among old growth wood, so it is possible that the exposure levels in our study were not sufficient to observe an effect.”*

2- Why did not the authors analyzed the stored bee bread, wax combs or honey from experimental colonies as they would be very sensitive to any volatile components.

**Author response:** The reviewer is correct that we did not analyze the hive matrices; rather, we focussed on assessing physiological impacts on individual bees (i.e. expression of detoxification enzymes and other proteins) as well as important indicators of overall colony health (i.e. adult population, brood area, colony weight, *Varroa* abundance, and overwintering survival). This is not an exhaustive list of things that could have been measured, but we consider these variables to be among the most important indicators of wellbeing. Bee bread, wax, and honey are of course essential components of the colony, and it is perhaps possible for some volatiles to infuse these products; however, if this occurs, it would only be relevant if it then impacts individual or colony health parameters. In our view, assessing these hive products would be worthwhile if we observed individual- or colony-level effects of wood type, as it would have provided information on the mechanism of the effect. However, since we observed no effect of wood type, there is no apparent mechanism to be determined, and if there are any changes in hive matrices induced by wood type, the effect appears to be benign. We do, however, think it is important to acknowledge that there are other parameters which we did not measure in our study, and we now mention this in the first paragraph of the discussion (see the new sentence quoted in response to point #1).

3- Is the Western red cedar (Thuja plicata) wood is the only type of cedar that is used in North America to manufacture hives.

**Author response:** Unfortunately, there is no database or survey information of wood types used in North American bee hives. We have been unable to confirm any wood types used in commercially available equipment other than red cedar and pine, but because “cedar” may colloquially refer to other species it is possible that species other than *Thuja plicata* are occasionally used. This is in part why we ended the discussion with the following sentence, to acknowledge that additional species may warrant further testing:

*“While our data do not reveal any positive or negative effects of western red cedar, there are many species of trees referred to as “cedar,” and each one should be tested to verify that it is benign (or beneficial) before being used on a large scale.”*

4- As most volatile components are affected by the time authors should specify the period of cedar wood storage (as row material and hives) before using in the experiment.

**Author response:** This is a good point and we have now added this information to the methods (line 104):

*“The lumber was allowed to dry for two months before constructing the beehives, and honey bees were introduced to the hive boxes three months later, when the wood was still highly aromatic.”*

5- As mentioned before that cedar hives are better isolated, then the honey area in combs should have been measured to ensure that.

**Author response:** We assume that the reviewer means “insulated”, rather than isolated. We are still not entirely sure how measuring the honey area would ensure that a hive is more or less insulated than another (during the summer, bees will store the same amount of honey in a cavity, regardless of the insulation of the walls), but we note that we did measure colony weight, which is predominantly impacted by stored honey.

6- if there any behavioral changes in the colonies, it should be mentioned especially in the forage and workers policing

**Author response:** We did not measure behaviours as part of this study; rather, we focused this work on individual and colony health outcomes as listed in our response to point #2. We are unsure of what is meant by “workers policing”, as this refers to a highly specific behaviour whereby workers detect and remove eggs laid by other workers. The direct relevance of this behavior to our study on hive wood types is unclear to us. Regardless, we do think that mentioning the fact that we did not measure specific behavioural changes in this study is worthwhile, and we have added this caveat to the discussion (see changes quoted in our response to point #1, where this is specified).
